# The working lives of neuronal 5-HT_2A_ receptors from the outside in

**DOI:** 10.1039/d6cb00014b

**Published:** 2026-05-07

**Authors:** Aneesah Jabar, Mikayla Pinto, Terence E. Hébert

**Affiliations:** a Department of Pharmacology and Therapeutics, McGill University Montreal Quebec H3G 1Y6 Canada terence.hebert@mcgill.ca

## Abstract

5-HT_2A_ receptors have been shown to play critical roles in regulating neuronal signalling networks and brain plasticity. They have become important targets for treatment of resistant forms of mental health disorders such as depression, anxiety and post-traumatic stress disorder. Like many G protein-coupled receptors (GPCRs), signalling downstream of 5-HT_2A_ depends on where the receptor is in the cell. Events and signalling pathways modulated by the cell surface pool of receptors may be distinct from those regulated by an internal pool of receptors. In this review, we parse out how the cell organizes and regulates this location bias and explore approaches to target the distinct receptor pools pharmacologically.

## Abstract 2

Signalling downstream of 5-HT_2A_ receptors may be driven by biased or location-biased ligands that will modulate the nature and kinetics of signals that may help discriminate between hallucinogenic and non-hallucinogenic receptor responses.

## Graphical abstract

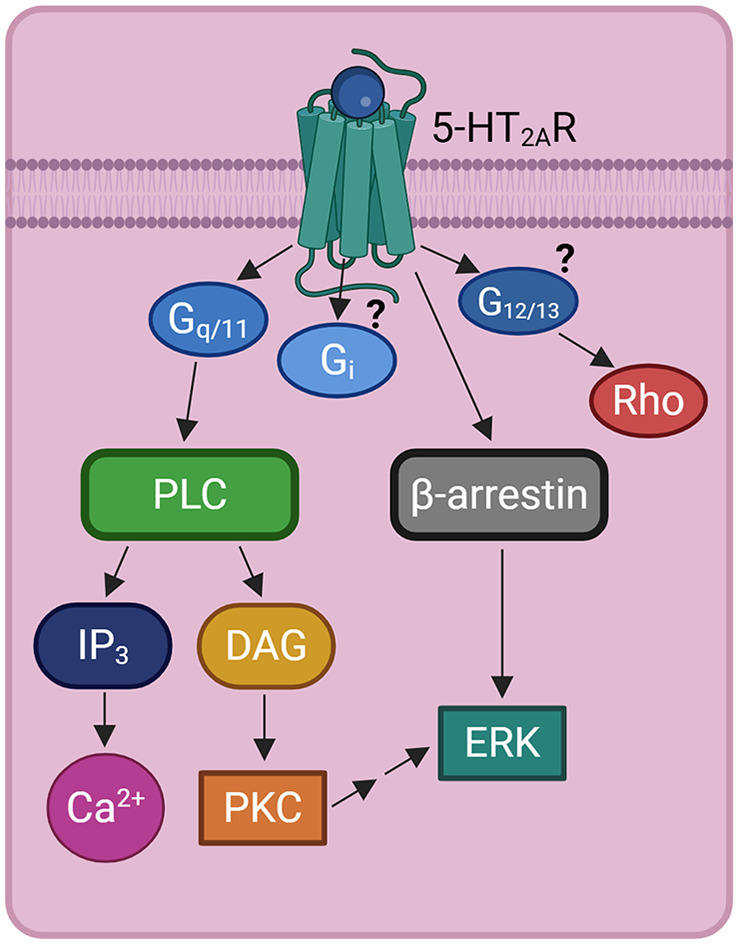

## 1. Introduction

G protein-coupled receptors (GPCRs) are transmembrane receptors that signal primarily through heterotrimeric G proteins and β-arrestin. They are expressed ubiquitously throughout the body and regulate numerous physiological functions, so it is not particularly surprising that GPCRs are targets for more than 30% of FDA-approved drugs.^1^ The human genome encodes over 800 GPCRs, and approximately 370 are non-sensory GPCRs.^1^ Most of these non-sensory receptors are present in the brain, including members of the serotonin (5-hydroxytryptamine or 5-HT) receptor family.^1,2^ Although serotonin is primarily produced in the gastrointestinal tract, its function as a neurotransmitter depends on its synthesis in the brainstem.^3^ In serotonergic neurons of the raphe nuclei, tryptophan is hydroxylated to 5-hydroxytryptophan and subsequently decarboxylated to synthesize 5-HT.^3,4^ Following its synthesis, serotonin is stored in these neurons, which send ascending projections to the forebrain and descending projections to the medulla and spinal cord.^3^ The release of serotonin at synaptic terminals modulates various regions of the central nervous system through 5-HT receptors.^3^ Due to their widespread distribution and established role in depression, anxiety and schizophrenia, these receptors stand out as promising drug targets.^5^ The 14 subtypes of 5-HT receptors are grouped into seven classes (5-HT_1–7_), all of which are GPCRs except for 5-HT_3_.^6^ Of relevance here, the 5-HT_2A_ receptor is distributed across several brain regions but is most densely expressed in pyramidal neurons and in γ-aminobutyric acid (GABA) interneurons in layer 4/5 of the pre-frontal cortex.^7–10^ Currently, this receptor is targeted by several antidepressants (trazodone and mirtazapine) and atypical antipsychotics (aripiprazole, risperidone and olanzapine), which predominantly act as receptor antagonists.^11^ In contrast, recent research has highlighted 5-HT_2A_ receptor agonism as a mechanism underlying psychedelic-assisted therapies, which has garnered attention for its potential to elicit rapid and sustained clinical benefits for treatment-resistant neuropsychiatric conditions.^12–19^ This review focuses on the complexities in understanding psychedelic signalling *via* the 5-HT_2A_ receptor and highlights spatiotemporal signalling as a promising avenue for future research.

## 2. A shift towards psychedelic-assisted therapy

Much of what we understand about the effects of psychedelics on the body and mind originates from their long-standing spiritual and healing use by indigenous peoples throughout the Americas.^20–22^ Subsequent studies in the 1950s and 1960s suggested that psychedelics were psychomimetic and could be used to study neuropsychiatric diseases; they have since been investigated for their potential to treat several mental illnesses.^23–27^ Serotonergic psychedelics primarily act through 5-HT_2A_ agonism to alter perception, thought, and mood. These hallucinogenic effects ultimately led to their prohibition in the 1970s. More recently, there has been renewed interest in psychedelic therapy for treatment-resistant neuropsychiatric disorders, as it may address some limitations of the existing treatments. On average, classically prescribed antidepressants take 3–6 weeks before the onset of therapeutic benefits; even then, at least 30% of diagnosed individuals remain treatment resistant.^11,28^ Furthermore, several early-phase clinical trials of psychedelic-assisted therapy have demonstrated rapid, promising therapeutic outcomes, some of which can last up to 12 months.^14,15^

Psychedelic-assisted therapy involves administering a controlled dose of a psychedelic, typically 25 mg of psilocybin, combined with several hours of psychotherapy before, during and after the session.^21,29,30^ Observational studies of recreational psychedelic use in the broader population, as well as evidence from small-scale trials, indicate that these compounds exhibit a favourable safety profile with low abuse potential.^20–22,31–33^ While commonly reported adverse effects such as nausea and headache are typically described as mild symptoms,^34^ we cannot ignore psychedelic agonism at 5-HT_2B_ receptors, which represents a more serious concern due to its association with cardiac valvulopathy.^35,36^ Despite the accumulating evidence supporting the development of psychedelic-assisted therapy, its adoption as a standard treatment remains limited due to unresolved concerns about the hallucinations or subjective effects. Amid ongoing debate regarding the necessity of hallucinations for clinical improvement, acute subjective psychedelic experiences appear to be therapeutically meaningful, as mystical experience questionnaire (MEQ) scores can predict psilocybin treatment success.^37,38^ The MEQ comprises four areas to assess the acute effects of psychedelics: mystical experience, positive mood, transcendence of time and space, and ineffability.^37^ Likewise, the historic use of psychedelics in community sessions highlights the dependence of context and setting on therapeutic outcomes. It was shown that intersubjective experiences in community psychedelic practice improve psychological well-being and social connectedness.^39^ The importance of group experience was equally noted in a pilot study involving indigenous participants. In this study, ketamine-associated experiences paired with a community setting was necessary for participants to break harmful patterns and to connect with their inner selves.^40^ Therefore, a community setting and guided sessions remain crucial factors in therapeutic psychedelic practices, in part, why therapy requires hours of clinical supervision. Regardless of the potential therapeutic relevance, the subjective effects of psychedelics increase costs and reduce accessibility, thereby impeding their widespread clinical development. Moreover, the unpredictable nature of hallucinations among individuals has led to strict exclusion criteria in psychedelic trials, often omitting those with a family history of psychotic disorders—who frequently have comorbidities of depression and other conditions.^13,29,41–43^ Therefore, a clearer understanding of whether it is possible to separate hallucinogenic from therapeutic effects will depend on mechanistic studies.

### 2.1 Hallucinations require 5-HT_2A_ receptors

As hallucinations remain a prominent concern in psychedelic therapy, it is important to understand their mechanisms of action to clarify how these compounds alter conscious experiences. Serotonergic psychedelics can broadly be divided into three chemical classes: tryptamines, ergolines and phenethylamines (Table 1). While this structurally diverse group of compounds has affinity for multiple serotonin, dopamine, histamine and adrenergic receptors, it is generally accepted that the subjective effects depend on 5-HT_2A_ receptor activation.^36^ Radioligand binding experiments support the interpretation that 5-HT_2A_ receptor activation by psychedelics leads to hallucinations.^44^ Further evidence supporting this includes the loss of hallucinogenic effects in 5-HT_2A_ rodent knockout models^45^ and the attenuation of subjective effects by 5-HT_2A_ antagonists in both rodents and humans.^46–49^ That said, rodent studies rely primarily on the head-twitch response (HTR) as a proxy for hallucinogenic effects in humans.^50^ Nevertheless, as a surrogate for hallucinations, the HTR has several limitations. Compounds from various drug classes that are not known to be hallucinogenic in humans can elicit a rodent HTR. These include select benzodiazepines, the cannabinoid CB_1_ antagonist SR 141716A, some 5-HT_1A_R antagonists, [Met]enkephalin, and carbachol.^51^ Additionally, the activation of 5-HT_2A_ receptors across several brain regions can produce a HTR, obscuring which specific regions are necessary for generating these responses.^51^ Notably, relying on HTR alone as a measure of hallucinogenic potential overlooks higher-order effects often altered in humans, such as attention and cognition. To track such alterations due to hallucinogens in rodents, the addition of the signal detection task or the 5-choice serial reaction time test may be beneficial.^52^ Taken together, most studies to date show 5-HT_2A_ as necessary for the hallucinogenic effects of psychedelics, but its precise role in mediating therapeutic effects is less understood.

**Table 1:** 5-HT_2A_ receptor ligand properties. 5-HT_2A_ receptor ligand hallucinogenic potential, partition coefficient, chemical class, and chemical structure. Partition coefficients (*X* Log *P*3) computed using *X* Log *P*3 3.0 (PubChem release 2025.04.14) for serotonin, LSD, 2-Br-LSD, lisuride, psilocin, DMT, 5-MeO-DMT, TBG, volinanserin, and bufotenine, (PubChem release 2025.09.15) for ariadne, ketanserin, and pimavanserin, (PubChem release 2024.11.20) for NBO, and (PubChem release 2021.10.14) for DOI. Partition coefficients (*X* Log *P*3-AA) computed for NBO, TBG, volinanserin, ketanserin, and pimavanserin. All chemical structures were obtained from PubChem

Drug	Hallucinogenic potential	Human or rodent study	Partition coefficient (Log *P*)	Chemical class	Chemical structure
Serotonin	No	Endogenous reference ligand	0.2	Tryptamine	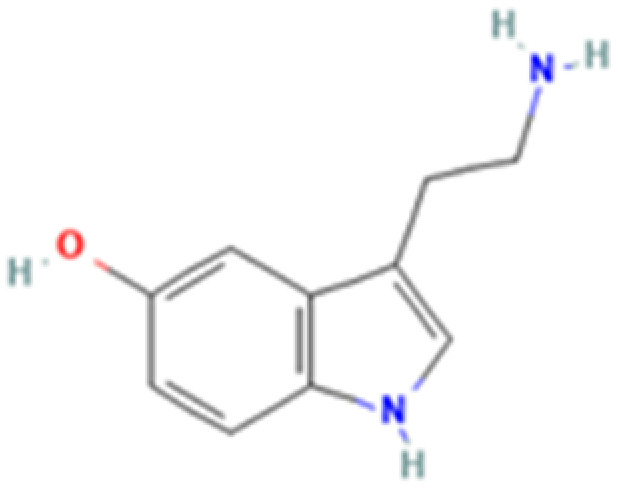
Psilocin	Yes	Human^181,182^	2.1	Tryptamine	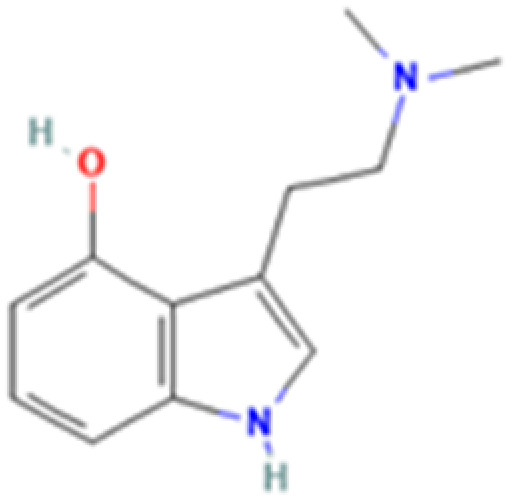
LSD	Yes	Human^183^ and Rat^85^	3	Ergoline	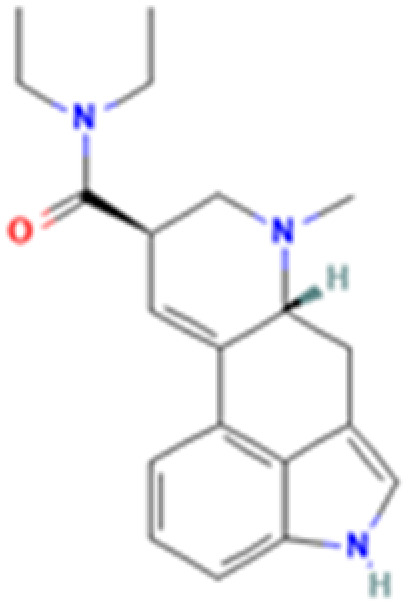
2-Br-LSD	No	Human,^77^ and Rat^67^	4	Ergoline	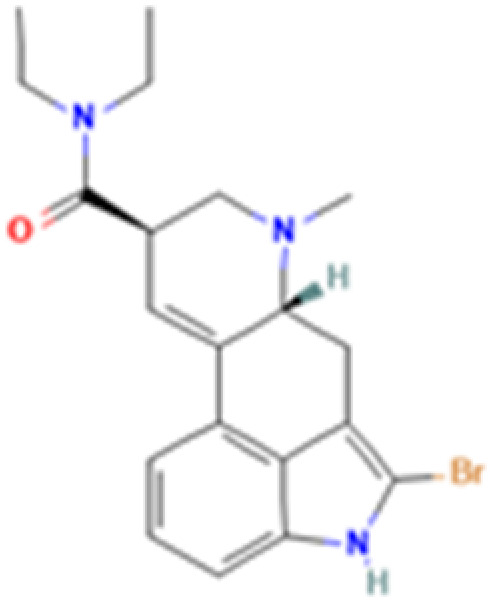
Lisuride	No	Human^184^ and Rat^85^	2.7	Ergoline	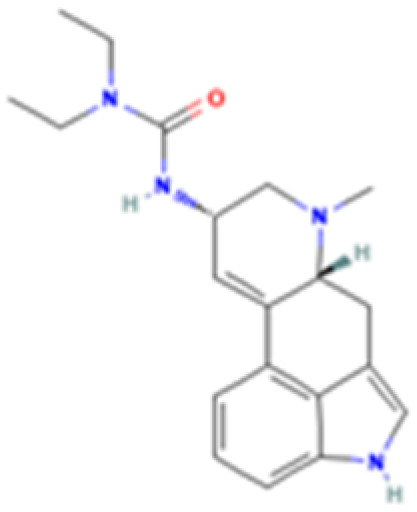
Ariadne	No	Human and Rat^75^	2.8	Phenethylamine	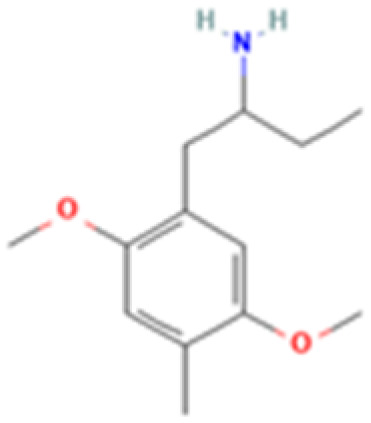
25CN-NBOH (NBO)	Yes	Mouse^185^	3.1	Phenethylamine	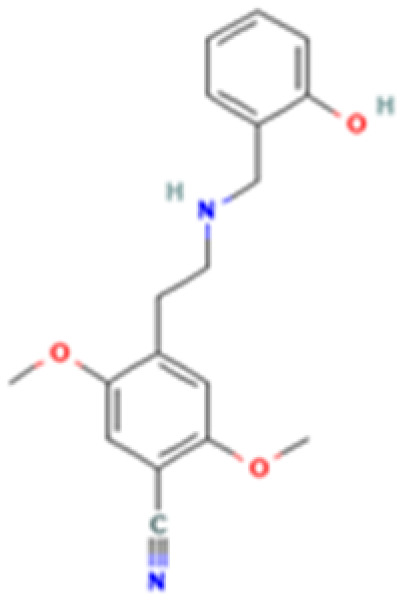
DOI	Yes	Rat^186^	2.5	Phenethylamine	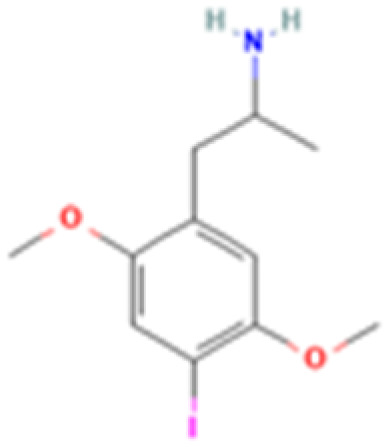
*N*,*N*-Dimethyltryptamine (DMT)	Yes	Human^187^	2.5	Tryptamine	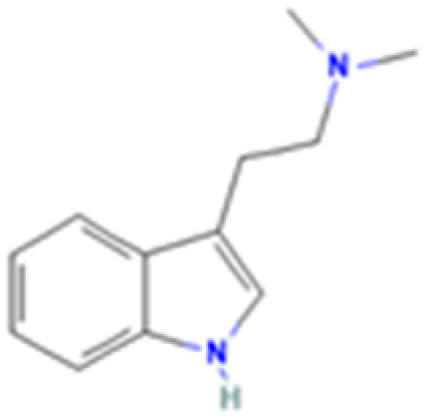
5-MeO-DMT	Yes	Human^188^ and Mice^189^	1.5	Tryptamine	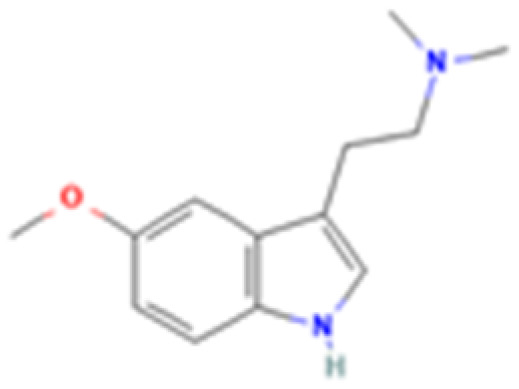
Bufotenine	Yes	Human and Rat^190^	1.2	Tryptamine	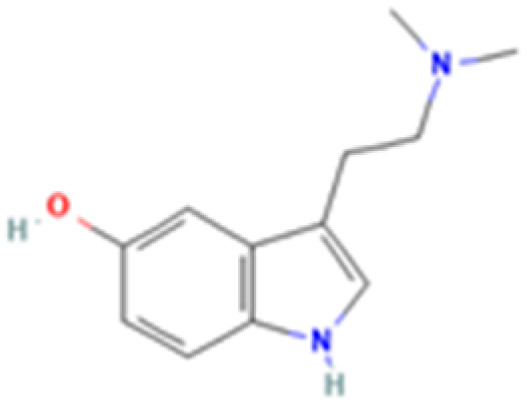
Tabernanthalog (TBG)	No	Rat^74^	2.4	Tryptamine	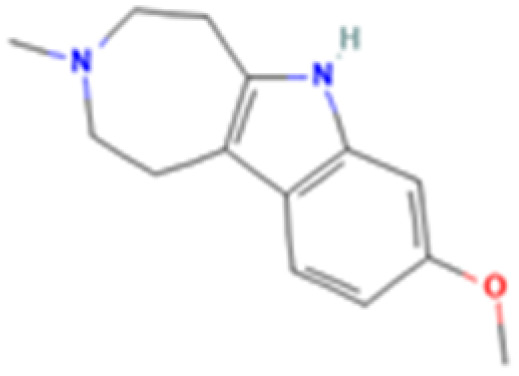
Ketanserin	No	Human^191^ and Rat^192^	2.6	Quinazoline	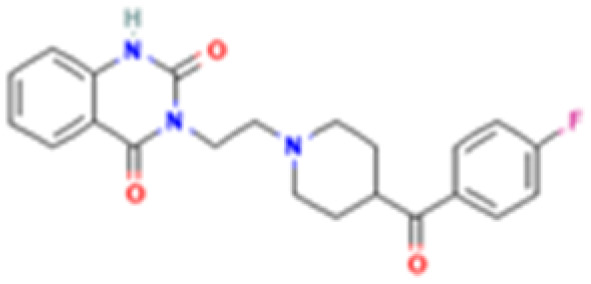
Volinanserin (MDL100907)	No	Rats^47^	3.9	Piperidine	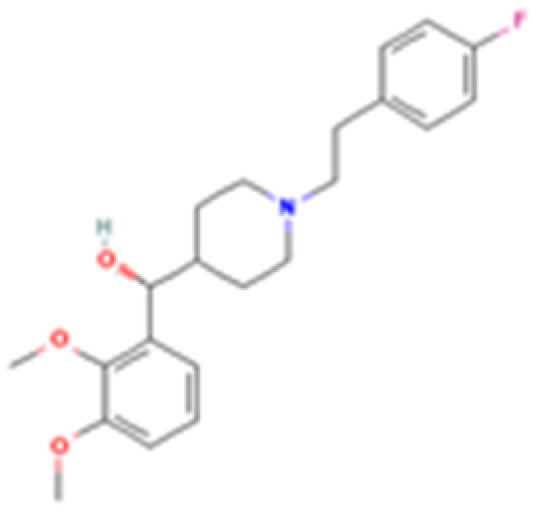
Pimavanserin	No	Human^193^	4.5	Urea	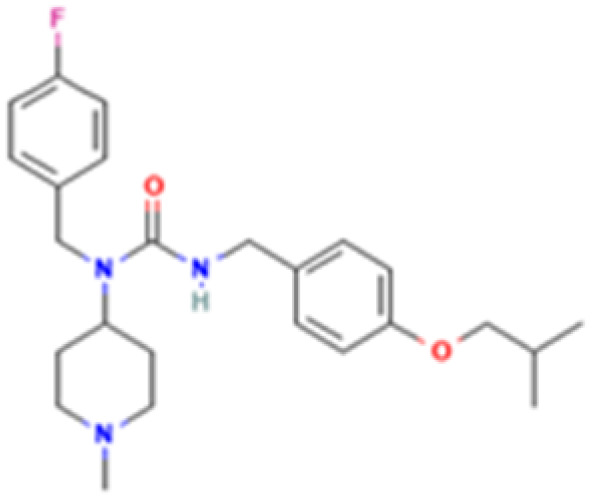

### 2.2 5-HT_2A_-mediated therapeutic effects

Psychedelics are under investigation for treating several conditions such as depression, anxiety, obsessive-compulsive disorder, substance use disorder, and post-traumatic stress disorder. Besides the monoamine hypothesis, one underlying theory in depression is the presence of cortical atrophy and reduced neuronal connections.^53,54^ Given that reduced dendritic growth is a common feature of several neuropsychiatric conditions, enhancing dendritic plasticity may represent a unifying mechanism of psychedelic therapy, consistent with the evidence that their therapeutic effects arise from promoting neuroplasticity and the formation of new neural connections.^55^ Neuroplastic changes similarly accompany the chronic use of classic antidepressants and the rapid-acting *N*-methyl-d-aspartate (NMDA) receptor antagonist ketamine.^56–61^*In vitro*, the therapeutic readout of psychedelics is typically measured by quantifying dendritic growth using Sholl analysis,^62^ while *in vivo*, it is evaluated using behavioural paradigms such as a forced swim test, sucrose preference test and tail suspension test.^63^ Much like our knowledge of the implications of hallucinogenic effects and 5-HT_2A_, the association between 5-HT_2A_ and therapeutic outcomes has also been demonstrated by antagonist and knockout studies.^64–69^

While psychedelics can induce neuroplasticity in brain regions that lack 5-HT_2A_,^66^ overwhelming evidence indicates that 5-HT_2A_-mediated plasticity in the prefrontal cortex (PFC) is critical for therapeutic effects. Still, a direct causal link between 5-HT_2A_ receptor signalling, structural plasticity, and behavioural outcomes remains unconfirmed. To address this issue, Shao and colleagues demonstrated that psilocybin increased spine density in both pyramidal tract and intratelencephalic neurons within the medial frontal cortex; however, only plasticity of pyramidal tract neurons was necessary for improvements in stress-related behaviours.^68^ To establish a direct connection between structural plasticity and 5-HT_2A_ signalling, it was shown that a selective 5-HT_2A_ knockout in pyramidal tract neurons abolished psilocybin-induced spine growth.^68^ Furthermore, in mice, the localized deletion of 5-HT_2A_ in the medial frontal cortex prevented psilocybin from reducing stress-driven phenotypes.^68^ In another study, tabernanthalog (TBG), a non-hallucinogenic analogue of 5-MeO-DMT, was shown to rely on 5-HT_2A_ for both dendritic spine growth in layer 5 pyramidal neurons and antidepressant-like effects in the tail suspension test.^64^ Consequently, the selective photoablation of TBG-induced dendritic spines in the prefrontal cortex abolished antidepressant-like responses.^64^ These approaches help to demonstrate the relation between 5-HT_2A_-mediated cortical spinogenesis and the behavioural effects of these psychoplastogens.

Generally, psychedelics are thought to exert their therapeutic effects by driving structural and functional neuroplasticity. Neuroplasticity is a broad term which can encompass neuritogenesis, dendritogenesis, spinogenesis and synaptogenesis. To understand whether these changes are consistent across studies and compounds, it may be beneficial to report several elements of structural and functional neuroplasticity in response to serotonergic psychedelics.^70^ Similarly, quantifying overall dendritic spine density alone overlooks changes in finer dendritic spine structural features, commonly grouped into four morphological categories: filopodia, thin, stubby, and mushroom. Due to their large size and stability, mushroom spines are often considered more mature and long-lasting compared to the rest.^71^ Although stubby spines are the second-largest subtype, they are still regarded as immature structures and believed to transition into more mature spine types.^65,71,72^ In contrast, thin spines form immature, highly plastic synapses that can strengthen with activity and are thought to participate in learning processes.^65,72^ The presence of filopodia-like structures peaks early in development, and they represent the smallest and most dynamic protrusions, with lifetimes lasting only up to a few hours.^72^ Hence, the characteristics and dynamics of dendritic spines represent unique stages of plasticity and may be functionally distinct regarding behavioural outcomes in psychedelic therapy. One study demonstrated that in a 5-HT_2A_-dependent manner, DOI-treated mice displayed increased cortical dendritic density by upregulating stubby and thin dendritic spines but not mushroom spines.^65^ In a separate experiment yielding similar results, DMT, LSD and DOI also favoured the growth of thin and filopodia spines over mushroom spines.^70^ Additional long-term assessments are required to understand whether the duration of structural neuroplasticity is necessary for the rapid or long-term behavioural changes. For instance, psilocybin and 25CN-NBOH give rise to persistent functional plasticity and antidepressant-like effects 12 weeks post-administration, without sustained structural plasticity at the same timepoint.^73^ Currently, these subtle details of spinogenesis are less understood with respect to 5-HT_2A_-mediated structural plasticity. Therefore, it may be more informative to examine changes in dendritic spine populations and their distribution, along with their respective rates of elimination and formation. Perhaps this approach will provide a more granular understanding of long-term behavioural changes than the total spine number alone. While neuroplasticity is a common readout for predicting therapeutic effects of 5-HT_2A_ agonists, it is ultimately an indirect surrogate, often assessed outside of the relevant circuit-level context. Integrating long-term measures of neuroplasticity in combination with *in vivo* behavioural outcomes may strengthen the correlation to therapeutic outcomes. Overall, the causal chain of events from receptor activation, precise signalling mechanisms, dendritic spine growth and therapeutic benefit must be further understood and likely to differ depending on the specific psychedelic examined.

## 3. The diversity of 5-HT_2A_ agonism: partial agonism *versus* biased signalling

An abiding mystery is that serotonin, among other 5-HT_2A_ agonists, is not perceived as hallucinogenic in humans. In fact, several studies have shown that non-hallucinogenic 5-HT_2A_ agonists, with varying selectivity, such as lisuride, ariadne, and 2-bromo-LSD can mediate several therapeutic effects, either through neuroplastic changes or behavioural paradigms in pre-clinical trials.^67,74–78^ This distinction has prompted several groups to explore whether it is possible to develop 5-HT_2A_ agonists that retain therapeutic effects with minimal hallucinogenic activity. Characterizing psychedelic signalling may clarify which pathways mediate hallucinogenic *versus* therapeutic properties.

A single GPCR can couple to multiple G proteins and trigger a number of distinct intracellular signalling cascades, ultimately driving gene transcription and translation to shape cellular behaviour and function. When serotonin binds to 5-HT_2A_, guanosine diphosphate (GDP) is exchanged for guanosine triphosphate (GTP), thus leading to the dissociation of the Gα_q/11_ subunit from Gβγ subunits. Gα_q/11_ activates phospholipase C (PLC), which cleaves the membrane phosphatidylinositol 4,5-bisphosphate (PtdIns(4,5)P2 or PIP_2_) to subsequently produce inositol 1,4,5-triphosphate (IP_3_) and diacylglycerol (DAG). IP_3_ binds to receptors on the endoplasmic reticulum, permitting Ca^2+^ release from the endoplasmic reticulum into the cytoplasm. Ca^2+^ and DAG are protein kinase C (PKC) activators; both Ca^2+^ and DAG are required for the activation of α, β and γ PKC isozymes, whereas novel PKC isozymes (δ, ε, η and θ) only depend on DAG. PKC activation leads to extracellular signal-regulated kinases 1/2 (ERK 1/2) through the mitogen-activated protein kinases (MAPK).^79^ Consequently, active, ligand-bound receptors are phosphorylated by GPCR kinases (GRKs), which subsequently recruit β-arrestin to desensitize the initial wave of signalling.^80^ Furthermore, β-arrestin can mediate receptor internalization, which can ultimately lead to receptor recycling and degradation or, importantly, elicit a second wave of signalling.^80,81^

Two major explanations posit why some 5-HT_2A_ agonists are hallucinogenic while others are not: functional selectivity and partial agonism. Functional selectivity, or ligand bias, commonly refers to a ligand's preferential activation of G protein- or β-arrestin-driven pathways.^80^ This may include a given receptor's ability to couple to G proteins beyond those initially identified as receptor-coupled. In the case of 5-HT_2A_, psychedelics have been proposed to involve activation of Gα_i/o_ proteins in addition to Gα_q/11_ alone.^45,82–84^ If some psychedelics are indeed biased, it may be possible to design drugs that selectively activate or avoid pathways differentially associated with certain physiological outcomes. *In vivo*, biased agonism may underlie some psychedelic-like behaviours observed with LSD and not with lisuride.^45,67,85–87^ Various studies describe LSD as a β-arrestin-biased ligand,^87–89^ whereas lisuride, a structurally similar non-hallucinogenic ligand, is classified as G protein biased.^90^ Although this distinction does not apply to all psychedelic and non-psychedelic analogues, LSD and lisuride are frequently used as benchmark comparators.^85^ β-arrestin 2 knockout mice treated with LSD showed reduced or no behaviours associated with psychedelic action, including measures of HTR, pre-pulse inhibition and motor activities, indicating that these behaviours at least partially depend on β-arrestin 2.^87^ These findings have been challenged by other attempts to synthesize biased 5-HT_2A_ agonists.

Based on a second binding mode observed in serotonin and psilocin, one group synthesized β-arrestin-biased 5-HT_2A_ ligands, with IHCH-7079 and IHCH-7086 being the two most potent ones.^91^ Interestingly, these compounds did not trigger a HTR but retained efficacy in alleviating depressive-like behaviours, as shown by tail suspension and forced swim tests.^91^ These findings align with recent studies demonstrating that β-arrestin-biased 5-HT_2A_ agonists fail to elicit HTR; instead, HTR positively correlates with 5-HT_2A_-driven, Gq signalling efficacy.^92^ Another structure-based screen of a virtual chemical library identified (R)-69 and (R)-70, agonists with greater 5-HT_2A_ selectivity compared to several classical psychedelics. While these G protein-biased compounds produced minimal HTR and did not disrupt prepulse inhibition compared to LSD, they nevertheless elicited antidepressant-like phenotypes in mice.^76^ Regardless, translating signalling bias measured in heterologous systems should be done with caution, as inconsistencies can arise from assays measuring proximal *versus* distal signalling events, and in the distinct stoichiometry of receptors, G proteins, and effectors noted between immortalized cell lines (*e.g.*, HEK 293 cells or SH-SY5Y cells), primary neurons, and *in vivo* models.^80^ Moreover, psychedelics are chemically diverse and exhibit a rich polypharmacology; thus, the ligand bias observed for one agonist, such as LSD, cannot be extrapolated to others without validation. Indeed, psychedelics belonging to separate chemical classes from LSD, such as psilocin or DMT, would stabilize slightly different 5-HT_2A_ receptor conformations, which may contribute to differences in signalling.^93^ The vast differences between psychedelics complicate decisions about whether prioritizing biased ligands is the most effective strategy for psychedelic drug discovery.

In contrast to functional selectivity, an alternative hypothesis suggests that non-psychedelic 5-HT_2A_ agonists simply behave as weak partial agonists.^67,75,91,92,94,95^ Biosensor-based studies indicate that bias toward Gα_q/11_ or β-arrestin does not consistently distinguish psychedelic from non-psychedelic agonists.^92,94^ Instead, non-hallucinogenic ligands are generally less efficacious than psychedelics in both Gα_q/11_-driven pathways (Gα_q/11_ dissociation from Gβγsubunits, IP_1_ accumulation, and increases in cytosolic Ca^2+^) and β-arrestin recruitment.^94^ Thus, partial agonism may explain why some 5-HT_2A_ agonists are non-hallucinogenic, but still, several psychedelics exist that are weak agonists. Some evidence suggests there is a threshold of Gα_q/11_ signalling needed to induce hallucinogenic effects, a higher threshold than what is required for therapeutic effects.^92^

## 4. 5-HT_2A_ receptors and location bias

**Fig. 1:**
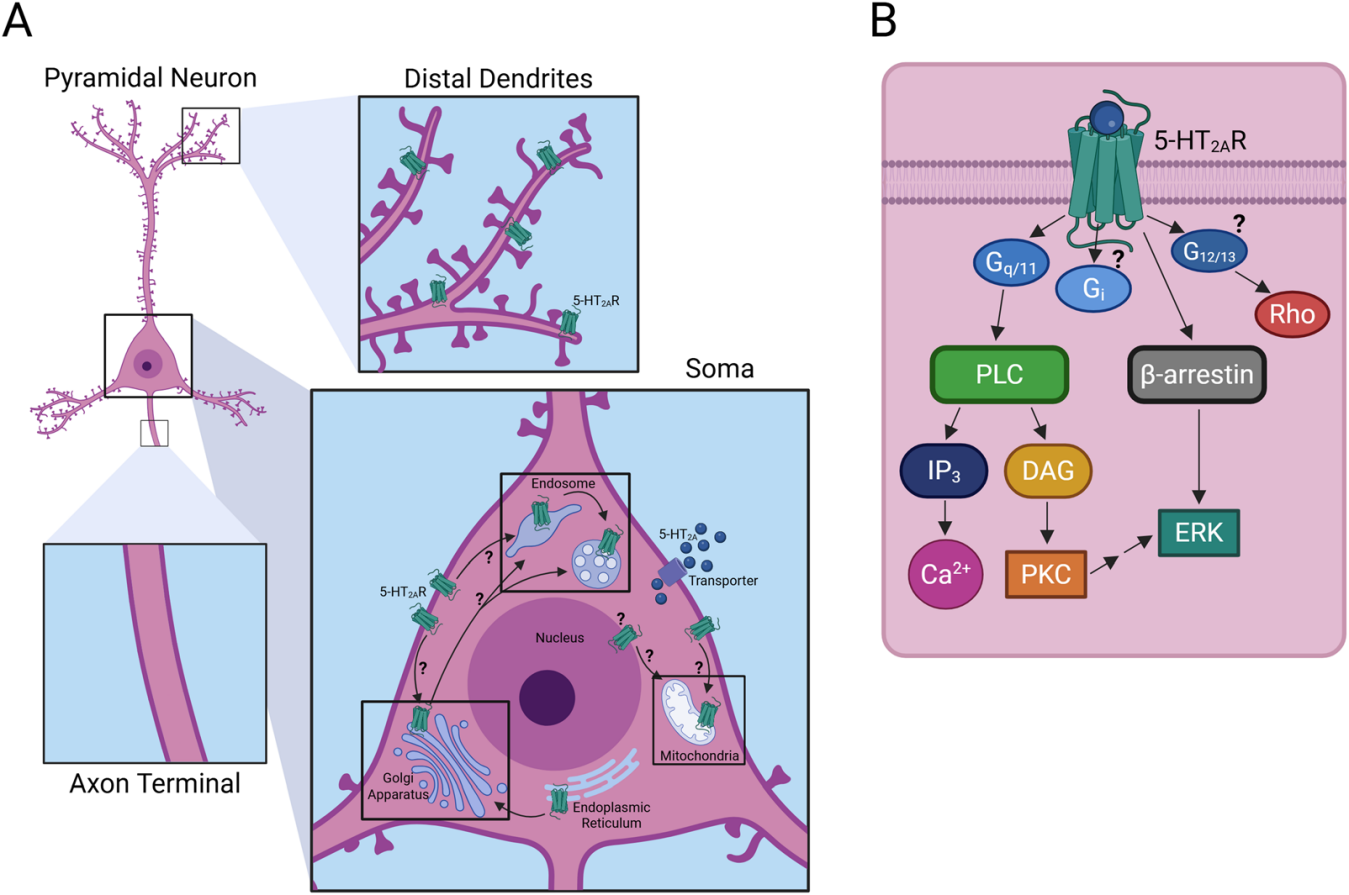
5-HT_2A_ localization and signalling. (A) In pyramidal neurons, 5-HT_2A_ receptors localize to distal dendritic regions and the soma while they are largely absent in the axon terminal. Experimentally known intracellular 5-HT_2A_ receptor pools exist in early and late endosomes, the Golgi apparatus, and mitochondria indicated by black boxes surrounding these locations. The localization in the nucleus and endoplasmic reticulum remains speculative. It is unknown whether intracellular receptor pools are a result of internalization from the cell surface or due to the biosynthetic pathway. Ligand internalization may be due to transporters or passive diffusion across the cell membrane. (B) Schematic of 5-HT_2A_ receptor signalling partners at the cell surface. These may differ across intracellular compartments possibly causing distinct signalling events such as recruitment of Gα_i_ and Gα_12/13_ by the 5-HT_2A_ receptor. Created with https://BioRender.com.^69^

Neither functional selectivity nor partial agonism provides a consistent overarching explanation for observed differences between hallucinogenic and non-hallucinogenic 5-HT_2A_ ligands. A common property among psychedelics, regardless of the chemical structure, is their ability to cross cell membranes. These properties differ drastically from the endogenous 5-HT_2A_ ligand serotonin (Table 1 and Fig. 1). The ability of a ligand to access distinct intracellular receptor pools highlights an important nuance regarding functional selectivity called location bias. This phenomenon is noted where the same receptor can initiate distinct signalling cascades when activated in different subcellular locations.^96^ While GPCRs are widely studied as plasma membrane-bound receptors, their localization to various subcellular compartments is gaining attention, including in the context of psychedelic signalling at 5-HT_2A_ receptors.^69^

### 4.1 Subcellular distribution of neuronal 5-HT_2A_ receptors

To study location bias, it is essential to understand the subcellular location of 5-HT_2A_ in a given cellular model, determine whether it is active at those sites, and assess how its downstream signalling cascades and gene expression profiles may vary based on location. However, many of these parameters are poorly characterized for 5-HT_2A_. Early studies using light and electron microscopy combined with immunocytochemistry revealed that across rat brain regions, most 5-HT_2A_ receptors could be detected in intracellular compartments.^7,8^ In several regions, including cortical neurons, 5-HT_2A_ receptors were localized to somato-dendritic and axonal areas.^7,8^ Within neurons, receptor expression tends to increase in distal dendritic regions compared to the soma but is largely absent in axon terminals and dendritic spines.^7,8^ While these studies did not identify receptor association with the Golgi apparatus, mitochondria, lysosome or endosome-like vesicles, 5-HT_2A_ was found to co-localize with microtubule-associated protein 1A, suggesting potential roles in neuronal plasticity and dendritic growth.^7,8^ While such techniques provide high spatial resolution, they may fail to capture transient or dynamic receptor distribution within specific subcellular compartments.

Considering that it is common to study GPCR signalling in immortalized cell lines, it is important to understand if location bias translates across experimental models. Immunocytochemistry and co-localization assays of fluorescently tagged proteins have revealed differences in the subcellular location between 5-HT_2A_ in primary neurons and HEK 293 T cells.^69^ Both endogenous and overexpressed 5-HT_2A_ in neurons colocalized with the Golgi apparatus to a greater extent than 5-HT_2A_ in HEK 293 T cells, see Fig. 1.^69^ Furthermore, 5-HT_2A_ also co-localized, to some extent, with mitochondria, early endosomes and late endosomes in both cell types.^69^ Understanding that 5-HT_2A_ receptors are not confined to the plasma membrane is only one piece of the puzzle; the overall contribution of internal receptors to cellular signalling remains largely unexplored.

### 4.2 Lipophilicity of serotonergic psychedelics and other 5-HT_2A_ ligands

Location bias has been thoroughly studied for several GPCRs, but it is a more recent addition in psychedelic research. Vargas and colleagues first showed that serotonergic psychedelics induce structural plasticity and antidepressant-like effects through internal 5-HT_2A_ receptor pools in cortical neurons.^69^ In that study, a series of approaches were implemented to reveal the contribution of intracellular receptors to neuroplasticity, including the use of membrane-impermeable 5-HT_2A_ antagonists to selectively block cell-surface receptors. Similarly, DMT and psilocybin lose their plasticity-inducing abilities when chemically modified with highly charged groups that prevent membrane permeation.^69^ Intriguingly, when serotonin is permitted to cross the plasma membrane through electroporation or SERT overexpression, it gains the ability to induce spinogenesis *in vitro*, and, *in vivo*, produces antidepressant-like effects.^69^ To date, this remains the only study to directly examine 5-HT_2A_ location bias. Their findings, as well as broader GPCR literature, emphasize the potential importance of ligand permeability in potentially therapeutic outcomes. The following sections explore possible implications for 5-HT_2A_ location bias in our understanding of psychedelic mechanisms.

Ligand permeability is generally described by the logarithm of the partition coefficient (Log *P*), which is determined by the ratio of the non-ionized ligand concentration in octanol to its concentration in water;^97^ several computational methods to estimate Log *P* values also exist.^98^ Alternatively, the distribution coefficient, Log D_pHX_, is a more informative measure as it accounts for both the pH of the intracellular compartment and the ionized and unionized forms of the compound.^97^ Among well-characterized 5-HT_2A_ agonists and antagonists, there is a spectrum of Log *P* values (Table 1). By correlating signalling profiles with permeability data, we can begin to disentangle contributions from internal *versus* surface receptor pools.

Molecular dynamics (MD) simulations provide an efficient computational approach to predict how alterations in a ligand's chemical groups affect permeability. Expectedly, MD simulations predicted that the protonation of tryptamine-based compounds DMT, bufotenine, 5-Meo-DMT and psilocin hinders their membrane partitioning.^98,99^ Such studies suggest that during membrane partitioning, psychedelics can alter membrane properties such as thickness, stiffness, and lipid order.^99–101^ These changes create thinner, loosely packed bilayers that may affect receptor dynamics, conformation, and ligand binding.^102,103^ Even among structurally similar ligands, subtle chemical differences influence how ligands prime the local receptor environment. For example, although bufotenine did not spontaneously partition the membrane, it was predicted to disrupt lipid membrane order to a greater extent than DMT and 5-MeO-DMT.^99^ Considering the structural diversity of psychedelics, ergolines and phenethylamines may perturb the membrane in distinct ways from tryptamines, as their chemical groups can differentially engage with lipid and phosphate groups.^101^ Several researchers have used MD simulations and artificial lipid membranes to explore how subtle chemical modifications can alter the permeability of tryptamine-class psychedelics. While serotonin is not generally permeable, simulations in artificial membranes indicate that it can partially insert into the bilayer and slightly reduce membrane stiffness and thickness.^100,101^ In contrast, DOI more strongly decreases lipid tail order and bilayer rigidity, promoting vesicular fusion.^101^ Therefore, psychedelics, or other lipophilic drugs, might reshape the membrane environment, tuning receptor behaviour simultaneously or prior to ligand binding. These tools not only strengthen our understanding of location bias but also provide valuable strategies for designing new chemical entities.^99,101,104^

It is likely that certain ligand–membrane interactions play a role in modulating GPCR signalling. Although phosphoinositides represent only a minor component of cell membranes, they are highly dynamic and essential for maintaining organelle identity, as well as GPCR signalling and trafficking.^103,105,106^ One of the earliest signalling events of 5-HT_2A_ after Gα_q/11_ subunit dissociation is the hydrolysis of PIP_2_ by PLC. Differences in membrane permeability among 5-HT_2A_ agonists may cause them to partition at different rates which in turn can alter membrane composition, possibly reflected by variations in PIP_2_ hydrolysis.^106^ In a study of PIP_2_ depletion in HEK 293 cells, both DOI (Log *P* = 2.5) and lisuride (Log *P* = 2.7) achieved similar sustained depletions of PIP_2_, but lisuride did so more rapidly.^106^ However, in primary cortical neurons, the magnitude and kinetics of the response were different; DOI induced a faster and greater PIP_2_ depletion than lisuride.^106^ Nevertheless, in both cell types, lisuride and DOI produced a greater PIP_2_ depletion than serotonin (Log *P* = 0.2), not accounting for PIP_2_ replenishment.^106^ Considering that lisuride and DOI are more lipophilic than serotonin, the previous results may reflect their similarities in membrane permeability. Understanding how psychedelics partition into membranes and influence PIP_2_ hydrolysis may advance our understanding of their signalling patterns. PIP_2_ has been shown to bind certain GPCRs, further stabilizing Gα protein interactions with the receptor.^103^ Additionally, PIP_2_ can facilitate interactions between β-arrestin and GPCR complexes; specifically, PIP_2_ influences β-arrestin orientation and interactions with the cell membrane.^107^ Therefore, it is reasonable to assume that a PIP_2_-mediated regulation of β-arrestin might differ in response to more lipophilic ligands, consequently influencing receptor internalization, desensitization or second-wave signalling. Testing both permeable and impermeable versions of the same ligand or using antagonists that vary in permeability (Table 1) to selectively block certain receptor pools may deepen our understanding.

Additional structural studies have demonstrated the role of plasma membrane lipids in modulating 5-HT_1A_ activity.^108^ Phosphatidylinositol 4-phosphate (PtdIns4P), the precursor of PIP_2_, binds between the receptor–G protein complex and promotes GTP hydrolysis, with PIP_2_ showing a similar but weaker effect.^108^ In parallel, membrane cholesterols enhance PtdIns4P binding and modulates aripiprazole–5-HT_1A_ interactions by stabilizing TM1 and TM7 of the receptor.^108^ Whether lipid-mediated interactions observed with other receptors also occur with psychedelics at 5-HT_2A_ receptors remains unknown, partly due to the complexity introduced by different cellular models. In human cells, lipid composition varies based on the developmental stage, disease state, and among different brain regions.^109,110^ Even within specified regions such as the cortex, lipid profiles are highly heterogeneous.^109^ Furthermore, membrane compositions in immortalized cell lines can significantly differ from those in primary cell cultures, further complicating our understanding of location bias.^111–113^ Therefore, more work is needed to determine how 5-HT_2A_ ligands prime the membrane and how the bidirectional relationship between the receptor and the phospholipid environment can shape signalling outcomes by different psychedelics.^114^

Ligand permeability can be advantageous in understanding location bias, however, designing experimental ligands based on permeability alone is unlikely to yield a physiologically relevant candidate. Previous studies highlight an important question about whether there is a favourable range of permeability or if specific ligand–membrane interactions are required for a given signalling outcome. Permeability beyond a certain Log *P* value might not meaningfully enhance a compound's pharmacological performance. Highly lipophilic ligands can become trapped within the plasma membrane, whereas less permeable ligands, when given enough time, may also cross the membrane and reach equilibrium.^115^ Likewise, the kinetic profile by which psychedelics and their analogues permeate the cell may alter membrane organization in a way that influences 5-HT_2A_ signalling. Additionally, since several non-hallucinogenic ligands display comparable Log *P* values to those of classical psychedelics (Table 1), these differences in hallucinogenic potential are unlikely to reflect a simple intracellular *versus* extracellular dichotomy. Instead, these processes are likely multifactorial, depending on the nature of compartment-specific signalling pathways; however, the extent to which these compounds retain canonical Gα_q/11_ and β-arrestin coupling *versus* alternate signalling partners remains unknown.

## 5. Avenues worth exploring to understand the 5-HT_2A_ location bias

While a more general distinction has been made between surface and internal receptor contributions to psychedelic outcomes, the precise subcellular contributions of 5-HT_2A_ signalling remain, to our knowledge, incompletely understood.^69^ Endosomal signalling from internalized agonist-activated receptors at the plasma membrane represents one facet of location bias, but several other GPCRs can also signal in the nucleus, mitochondria, and Golgi.^116^ Here, we use endosomal signalling as an example of how to approach signalling from internalized receptors. To study location bias, it is necessary to (1) determine if 5-HT_2A_ receptors in different compartments can reach active conformations and (2) understand the location-specific contribution to signalling.

### 5.1 Endosomal receptor signalling

As described earlier, upon receptor activation, GRKs phosphorylate the C-terminal tail of the receptor, promoting β-arrestin recruitment, which can both desensitize a response and facilitate clathrin-dependent receptor internalization.^80^ Receptor internalization does not always halt signalling; many G proteins (*e.g.*, Gα_q/11_, Gα_s_, and Gα_i/o_) and receptors (*e.g.*, β_2_-adrenergic receptor, dopamine D1 receptor and opioid receptors) continue to signal in endosomes, sometimes in ways distinct from their plasma membrane-bound versions.^117–124^ In several studies, GPCR endocytosis has resulted in sustained signalling of cAMP, ERK1/2, PKC and mTOR.^125,126^ This spatial shift in signalling may alter functionally relevant distal outcomes, as seen in the case of endosomal β_2_-adrenergic receptors, which can generate distinct transcriptional^123^ and translational^120^ profiles. In the case of Gα_s_-coupled receptors, there are several proposed mechanisms as to why endosomal signalling differs. First, endosomal receptors are increasingly distant from phosphodiesterases near the cell surface, while also in closer proximity to the nucleus, increasing the likelihood of cAMP signalling-dependent gene expression.^123^ Second, β-arrestin-mediated internalization can scaffold components of the MAPK pathway in proximity to endosomal receptors, thereby prolonging some signalling pathways.^125,127^ Notably, the conformation in which β-arrestin binds to GPCRs can facilitate different signalling outcomes. The “core” conformation, required for receptor desensitization, is where β-arrestin interacts with both the transmembrane core of the receptor and the phosphorylated C-terminal tail.^128^ In contrast, interactions through the “tail” conformation permit receptor internalization without desensitization.^128^ Thus, in some cases of GPCR internalization, β-arrestin remains bound to the C-terminal tail while the receptor core simultaneously remains available for G protein interactions.^125^ These interactions vary across GPCR classes, offering insight into which receptors are predisposed to prolonged signalling pathways. Class A GPCRs, such as 5-HT_2A_, are generally thought to form more transient interactions with β-arrestin, whereas class B GPCRs form more stable complexes due to longer C-terminal tails.^125^ For instance, in class B GPCRs, such as the parathyroid hormone 1 receptor (PTHR), sustained cAMP production from endosomes has been linked to the presence of β-arrestin–receptor complexes.^117,129^ But for the thyroid-stimulating hormone receptor, a class A GPCR, β-arrestin interactions are transient and, after internalization, are not thought to directly participate in prolonged cAMP signalling at the trans Golgi network.^117,130^ However, it is clear that the role of β-arrestin varies among receptors even within the same GPCR class;^131^ therefore, the interplay between 5-HT_2A_ and β-arrestin recruitment will have to be further studied in the context of spatiotemporal signalling from endosomes.

Several studies have shown that Gα_q/11_-coupled receptors can also elicit endosomal signalling, including the angiotensin II type 1 receptor, B_2_ bradykinin receptor, oxytocin receptor, thromboxane receptor, muscarinic acetylcholine receptor M_3_, and neurokinin 1 (NK1) receptor.^124,132^ Among these examples, signalling from specific compartments has resulted in unique physiological outcomes. Activation of the NK1 receptor in endosomes by substance P was shown to sustain spinal neuron firing and nociception.^132^ Subsequent targeting of endosomal NK1R by cholestanol-conjugated antagonists that facilitate internalization produced more effective maintenance of pain relief than plasma membrane-restricted NK1R inhibition, demonstrated by a reduced von Frey response in mice.^132^ Similarly, the PTHR can signal from both the plasma membrane and endosomes.^133^ To investigate the role of PTHR location on biological outcomes, one group used the spatially biased PTHR ligand, PTH7d, to prevent β-arrestin coupling to PTHR and maintain cAMP signalling at the cell surface but not at endosomes.^133^ The absence of endosomal signalling decreased the formation of the rate-limiting hydroxylase necessary for vitamin D production, highlighting the distinct physiological outcomes of PTHR endosomal signalling.^133^ Lastly, a recent pre-print highlighted the therapeutic relevance of the constitutively internalized human cytomegalovirus-encoded receptor (US28), which primarily signals through Gα_q/11_ from endosomal compartments.^134^ The study demonstrated that confining US28 to the plasma membrane led to the upregulation of oncomodulatory genes, as well as increased neutrophil chemotaxis and cell proliferation in glioblastoma spheroids. These findings suggest that plasma membrane Gα_q/11_ signalling drives oncogenic processes more strongly than endosomal signalling.^134^ Based on our knowledge of the previously mentioned Gα_q/11_-coupled GPCRs, it is worth examining whether 5-HT_2A_ also signals from endosomes or other endomembrane locations as has been reviewed for numerous other GPCRs.^116,135–137^

While conventional GPCR signalling is initiated at the plasma membrane, we have seen that in some cases, receptor internalization can result in sustained or second wave signalling events. However, for signalling to occur at separate compartments, it requires the presence of appropriate G proteins, effectors, and substrates. Currently, tools exist to detect Gα_q_ protein trafficking to endosomes as well as their activation.^124^ Regarding the angiotensin II type 1 receptor (AT1R), it was found that although Gα_q_ trafficked to endosomes independently of β-arrestin, endosomal Gα_q_ activation partially relied on the presence of β-arrestin.^124^ The same study also suggested that endosomal Gα_q_ activity is enhanced by prior activation at the plasma membrane and by β-arrestin-dependent AT1R internalization.^124^ Whether these findings are generalizable to other Gα_q/11_-coupled GPCRs like 5-HT_2A_ has yet to be determined.

Although Gα_q/11_ proteins and Gα_q/11_-coupled GPCRs are active at endosomes, the mechanisms by which they propagate their signals are unresolved, as some canonical substrates and effectors are missing in endosomes.^138^ The substrate for PLC, PtdIns(4,5)P2, is present in the plasma membrane but is essentially absent in endosomes. This change in lipid environment occurs during endocytosis when clathrin binds the AP2 adapter complex, creating a depletion in the enzyme that converts PtdIns(4)P to PtdIns(4,5)P2.^138^ Therefore, it is unlikely that DAG is produced in endosomes, and plasma membrane-derived DAG is not expected to diffuse readily between membranes. This is consistent with the inability of current detection methods to observe DAG in early endosomes.^124,138^ Although PKC has been identified in endosomes, most isoforms remain inactive without DAG.^138,139^ Thus, Gα_q/11_ activation at endosomes may initiate non-conventional pathways, either independently of PLC or *via* mechanisms that depend on prior activation at the plasma membrane. This could include the direct activation of endosomal adenylyl cyclase isoforms by calmodulin, ERK5 activation, and trafficking of active PKC to endosomes.^138,140^ Internal 5-HT_2A_ signalling may resemble plasma membrane signalling by activating pathways downstream of Gα_q/11_, or it may engage in separate pathways by coupling to alternative G proteins such as Gα_i/o_, or with a greater reliance on Gβγ and β-arrestin signalling in such compartments. In any case, the kinetics of signalling are likely to be altered.

### 5.2 Rho GTPases: an underexplored pathway in 5-HT_2A_ psychedelic action

An alternative signalling cascade of 5-HT_2A_ receptors may involve the activation of certain Rho family GTPases, which are found both at the plasma membrane and in intracellular compartments including the nucleus, endosomes and the Golgi apparatus.^141^ Rho GTPases are regulated by guanine nucleotide exchange factors (GEFs), which catalyze the exchange of GDP to GTP, and by GTPase-activating proteins which promote GTP hydrolysis.^142^ Rho GTPases act as molecular switches to mediate several roles including cytoskeleton organization, cell shape, microtubule dynamics, cell migration, and, notably, dendritic spine formation and synaptic plasticity.^142–144^ In *Xenopus laevis* tadpoles, Rac1 and Cdc42 mediate dendritic branch addition and retraction.^143^ Additionally, transamidation of Rac1 and Cdc42 by transglutaminase has been shown to produce transient dendritic spine enlargements following 5-HT_2A_/5-HT_2C_ stimulation by DOI in primary rat cortical neurons.^145^ Specifically, an alternative signalling cascade of 5-HT_2A_ receptors may involve RhoA.^146^ RhoA is an important part of dendritic branch elongation, with reduced RhoA activity promoting dendritic growth, a process that may be regulated by NMDA receptor activity.^143^ Furthermore, Rho GEFs can be activated by select G proteins, mainly Gα_12/13_ and, with a lower potency, Gα_q/11_, both of which may be coupled to 5-HT_2A_.^92,147,148^ This is exemplified by the proposed involvement of Rho in the 5-HT_2A_ receptor G_12/13_-coupled activation of PLA_2_.^83^ Additionally, p63RhoGEF, an effector of Gα_q/11_ found in the brain, enables Gα_q/11_ to activate RhoA, RhoB and RhoC.^147–150^ Evidently, RhoA activation by Gα_q/11_ can occur in a PLC and Ca^2+^-independent manner, suggesting a possible signalling pathway in the endosome where PLC is largely absent.^138,148^ However, p63RhoGEF, is predominantly localized to the plasma membrane and the related cytoplasmic RhoA effector GEFT has minimal activation unless artificially recruited to the plasma membrane.^149,151^ These observations suggest that Gα_q/11_-mediated Rho activation is likely influenced by GEF localization. Importantly, psychedelic-induced 5-HT_2A_ activation increases RhoA activity in HEK 293 cells.^152^ Given the diverse subcellular localizations and distinct physiological roles of Rho GTPases, together with evidence that Gα_q/11_ can signal from intracellular compartments, it is tempting to speculate that intracellular Gα_q/11_ engages Rho GTPases to promote dendritic growth and spine formation.^124,143^ Regardless, a deeper understanding of 5-HT_2A_-mediated Gα_q/11_ signalling through p63RhoGEF and Rho GTPases may provide insight into the mechanisms underlying psychedelic-induced plasticity.

### 5.3 Candidate ligands for intracellular 5-HT_2A_ receptors

In cortical neurons, the contribution of compartment-specific 5-HT_2A_ receptors is not fully understood, but collectively, the intracellular receptor pools promote neuroplasticity when activated by exogenous lipophilic psychedelics.^69^ Considering serotonin is hydrophilic, this raises the question of whether another endogenous ligand might modulate these receptors. Once serotonin is synthesized in serotonergic neurons of the raphe nuclei, it is packaged into vesicles by vesicular monoamine transporter 2 until it is later released into the synaptic cleft. Serotonergic neurons project widely throughout the brain and express active transporters such as serotonin reuptake transporters (SERT) to modulate serotonin levels. Since serotonin is protonated at physiological pH, it does not readily cross cell membranes; instead, it relies on SERT for uptake. SERT is highly expressed on presynaptic serotonergic terminals but less so on postsynaptic neurons, including those in the cortex.^4,153,154^ Nevertheless, we should not rule out serotonin, as several proposed mechanisms could allow it to reach internal 5-HT_2A_ receptor pools in these neurons.

SERT has a high affinity for biogenic amines, but serotonin uptake can still occur passively through low-affinity but high-capacity transporters, including organic cation transporters (OCT1–3) and plasma membrane monoamine transporters (PMAT).^4,155^ The broad expression of these transporters across the brain suggests they may permit serotonin entry into cortical pyramidal neurons, possibly even at low serotonin concentrations.^4,156^ Evidently, electron microscopy has revealed that OCT3 exists in the plasma membrane, mitochondrial, and nuclear pools of cortical neurons thereby providing an opportunity for serotonin to cross several membranes.^4^ In fact, the role of transporters in spatially compartmentalized GPCR signalling is not uncommon. For example, the activation of Golgi-bound dopamine D1 receptors depends on dopamine uptake *via* OCT2, and likewise, OCT3 may help permit norepinephrine access to the nuclear pool of β_1_-adrenergic receptors in astrocytes, driving nuclear protein kinase A (PKA) activity.^157,158^ Similarly, neuronal PMAT and OCT3 transport norepinephrine into the lumen of secretory vesicles, enabling activation of intracellular β_2_-adrenergic receptors, a mechanism proposed to contribute to long-term potentiation and synaptic plasticity.^159^ Therefore, modulating the expression or function of certain transporters may help clarify whether they permit serotonin to activate intracellular 5-HT_2A_. Evidently, the fate of serotonin once it enters postsynaptic neurons may be irrelevant to neuroplasticity, as it is likely to exist in low quantities and is subjected to degradation by monoamine oxidases. This is further supported by findings that serotonin-induced dendritic growth is observed only when SERT is overexpressed in specific neurons.^69^ Nonetheless, experimentally, little is known about whether, and to what extent, serotonin activates internal 5-HT_2A_ receptors in transporter overexpression systems. Instead, intracellular serotonin may function as an antioxidant within the mitochondria and regulate gene expression through the serotonylation of nuclear histones.^4^

Aside from serotonin, other endogenous ligands may activate intracellular 5-HT_2A_ receptors, including DMT. While DMT is well-recognized as the psychoactive component of ayahuasca, it is also naturally detected in human urine, blood, and cerebrospinal fluid.^160,161^ Whether DMT is synthesized in the human brain or is produced peripherally and crosses the blood–brain barrier remains uncertain. However, the rat brain can produce DMT at levels comparable to those of classical monoamine neurotransmitters and contains enzymes involved in its biosynthesis, though several synthetic pathways have been proposed.^162,163^ These findings suggest that DMT could be an endogenous ligand for intracellular 5-HT_2A_ receptors, although its physiological role is still under speculation.^164–166^

## 6. Perspectives

There is currently no consensus on whether the hallucinogenic and therapeutic effects of psychedelics can be mechanistically separated. Existing evidence does not clearly establish whether these subjective experiences are required for clinical benefit or represent unnecessary secondary effects. To this end, several frameworks have been proposed for understanding the mechanisms through which psychedelics induce each effect. Of these, partial agonism, biased agonism, and location bias are at the forefront. While generally accepted as non-hallucinogenic, high doses of lisuride, a partial 5-HT_2A_ agonist, have been shown to induce hallucinations in Parkinson's patients.^167^ This indicates a possible signalling threshold for hallucinations, such that partial agonism prevents hallucinogenic effects while higher doses overcome this threshold. Additionally, the synthesis of several β-arrestin or Gα_q/11_-biased 5-HT_2A_ agonists has resulted in contradicting physiological outcomes.^91,92^ Even so, partial agonism and biased agonism do not provide an all-encompassing explanation for the differences in hallucinogenic and non-hallucinogenic 5-HT_2A_ ligands. Therefore, location bias becomes an important nuance in psychedelic signalling profiles. As Vargas *et al.* have demonstrated, intracellular 5-HT_2A_ signalling mediates the neuroplasticity necessary for psychedelic therapeutic effects.^69^ Among the ongoing debates on the necessity of hallucinations for the therapeutic benefit, each of these frameworks may be crucial for elucidating how these effects can be separated.

Moving forward, we believe that the focus should be shifted towards understanding how location bias shapes psychedelic actions at 5-HT_2A_. To summarize our current understanding, the only direct evidence for 5-HT_2A_ comes from Vargas *et al.* (2023) in HEK 293 cells and primary cortical neurons. From colocalization and electron microscopy experiments, we know that 5-HT_2A_ is distributed throughout several compartments, yet precise signalling pathways inside specific subcellular compartments *versus* the cell surface receptor pool have not been experimentally determined. Although the remaining evidence for 5-HT_2A_ location bias is largely indirect, consistent findings from other GPCRs in heterologous and primary cells suggest that it may play a role in shaping 5-HT_2A_-mediated effects for psychedelic and non-psychedelic compounds.

As discussed, several intracellular signalling events are thought to mediate physiological responses that are not otherwise achieved by plasma membrane-bound receptors. Therefore, the intracellular pool of 5-HT_2A_ receptors may also regulate distinct physiological outcomes, influencing both therapeutic and hallucinogenic effects, representing an interesting area for future discovery.^69^ Intriguingly, serotonin appears to elicit the HTR *via* intracellular 5-HT_2A_ receptors, which merits further testing of other non-hallucinogenic ligands (Table 1) in similar assays to determine whether this subset of receptors is responsible for both therapeutic and hallucinogenic outcomes.^69^

The use of chemically modified ligands to spatially restrict psychedelics has enabled broad distinctions between internal and external receptors, yet much is unknown about location bias in psychedelic research. Moving forward, we must pinpoint the specific subcellular receptors and the signalling pathways attributed to psychedelic and non-psychedelic 5-HT_2A_ agonists. This includes making a distinction between actively signalling intracellular 5-HT_2A_ receptors *versus* receptors undergoing post-translational modifications and trafficking to the plasma membrane *via* the secretory pathway. Currently, several tools exist to delineate location-specific signalling events *in vitro*, including genetically encoded fluorescent nanobodies and mini-G proteins that recognize the active state of a GPCR.^119,158,168^ Biosensors confined to select subcellular compartments permit the recording of site-specific signalling events, as demonstrated using a CAAX motif or a FYVE domain that target the plasma membrane and endosomes, respectively.^169,170^ Similarly, we can leverage endocytosis inhibitors and chemically inducible dimerization strategies to probe the contribution of endosomal receptors to signalling outcomes.^123,168^ In a related approach, exploiting nuclear export and nuclear localization signals in biosensor constructs allows for a compartment-specific readout of several kinases, including ERK and PKA.^171,172^ Receptors present in the Golgi apparatus are markedly more difficult to study, given that they can house both internalized receptors from the plasma membrane and newly synthesized GPCRs. Additionally, the use of cycloheximide can block the 5-HT_2A_ biosynthetic pathway, which makes it possible to separate Golgi-localized receptor signalling.^173^ A combination of these tools has improved our knowledge about active GPCRs in endosomes (*e.g.*, β_2_-adrenergic receptor), the Golgi (*e.g.*, dopamine 1 receptor, opioid receptors, and thyrotropin receptor (trans-Golgi network)) and the nucleus (*e.g.*, angiotensin type II 1 and 2 receptors, metabotropic glutamate receptor, and β_1_- and β_2_-adrenergic receptors), to name a few.^116,119,122,158,168,169^ While these techniques have been extensively studied in immortalized cell lines, further investigation is warranted in primary neurons, cortical neuronal stem cells, and *in vivo*, particularly for 5-HT_2A_ receptors. Overexpression systems can result in altered receptor trafficking and distribution due to transfection conditions (*e.g.*, DNA concentrations, transfection efficiency, and translation rates) or the absence of scaffolding proteins such as postsynaptic density protein 95,^174^ leading to effects that cannot be recapitulated in physiologically relevant cell types.^69,130^ Furthermore, despite the high sequence homology between rat and human 5-HT_2A_ receptors, they still display marked differences attributed to their C-terminus.^173^ For instance, only the human 5-HT_2A_ receptor requires β-arrestin and GRK-2 for internalization, also taking roughly twice as long as the rat 5-HT_2A_ receptor to recycle post-internalization.^152,173,175^ This reveals an important caveat when comparing receptor signalling and trafficking data in different cellular systems in the literature.

Understanding the relationship between proximal 5-HT_2A_ signalling events, distal signalling, and subsequent gene expression also remains an important area of investigation. Very few studies have examined transcriptional profiles between hallucinogenic and non-hallucinogenic 5-HT_2A_ agonists,^45,176^ but whether the activation of 5-HT_2A_ in certain locations drives distinct transcriptome or translatome profiles is not known. Moreover, 5-HT_2A_ can form heterodimers with several receptors, including the dopamine D2 receptor and the metabotropic glutamate receptor 2 (mGluR2), further altering its signalling properties.^177–179^ 5-HT_2A_ dimerization represents a relevant aspect of psychedelic mechanisms, especially since these complexes alter receptor trafficking properties.^180^ Therefore, signalling profiles between hallucinogenic and non-hallucinogenic agonists, driven from different subcellular compartments, should be followed by functional assays to determine their impact on behavioural outcomes *in vivo*. This task becomes complicated because such effects likely occur at a circuit level, involving multiple receptors, genes and brain regions. From our current knowledge, the 5-HT_2A_ receptor is critical in mediating the therapeutic benefits of psychedelics in neuropsychiatric diseases. Several tools are at our disposal to discern between 5-HT_2A_ signalling biases, revealing that the diverse signalling at the cell surface and intracellular compartments represents a vast pool of knowledge waiting to be uncovered.

## Author contributions

A. J. wrote the primary draft; M. P. added to this draft and developed the figure and table. T. E. H. edited the draft and contributed to the writing of the final version.

## Conflicts of interest

There are no conflicts to declare.

## Data Availability

Since this is an invited review, there are no data to share.
